# MOVING BEYOND DISCRIMINATION: BUILDING A COALITION FOR LGBTQ+ OLDER PERSONS LIVING IN EAST TENNESSEE

**DOI:** 10.1093/geroni/igae098.3868

**Published:** 2024-12-31

**Authors:** Joseph Winberry, Sharon Bowland, Jennifer Smith, Namrata Mukherjee

**Affiliations:** University of North Carolina at Chapel Hill, Durham, North Carolina, United States; University of Tennessee, Knoxville, Tennessee, United States; University of Tennessee, Knoxville, Knoxville, Tennessee, United States; University of Tennessee Knoxville, Knoxville, Tennessee, United States

## Abstract

**Aims:**

Research with 50+ older members of the LGBTQIA+ community, conducted by Winberry (Forthcoming), aimed to identify important needs and healthcare gaps in services in East Tennessee. This poster describes establishing a coalition to address their complex needs and gaps.

**Methods:**

As part of a larger community-based participatory research project, a strategic plan created through interviews and focus groups with LGBTQIA+ older persons identified the type of partners needed to accomplish what this population wanted around aging services.

**Results:**

The coalition was formed in a three-step process. First, the earliest members identified organizations and individuals who could address participants’ needs: Culturally competent healthcare, services specifically for LGBTQIA+ people, social support, life planning, and caregiver services. Second, potential members who could address those needs were engaged. Those with personal ties or compelled by a social justice ethos around the LGBTQIA+ community were particularly interested in joining. Last, the role of the coalition members had to be negotiated, given limitations around staff, money, and time. Understanding exactly how members will contribute and when this needs to be renegotiated is essential to coalition success.

**Conclusion:**

The Aging Rainbow Coalition (ARC)—consisting of older members from the LGBTQIA+ community, the local Pride agency, the state flagship land grant university, and other aging services organizations—was established at the county Office on Aging. This poster on the coalition creation process provides details across the three steps of the creation process and highlights insights useful for researchers and practitioners.

